# Effectiveness of exercise on musculoskeletal function and clinical outcomes in patients with diabetic peripheral neuropathy: a systematic review and meta-analysis

**DOI:** 10.3389/fneur.2025.1610955

**Published:** 2025-08-29

**Authors:** Jin Yang, Li Li, Ting Ye, You Pu, Qian Yao, Jian Luo, Yunman Huang, Xianqin Zhang, Zheng Yang

**Affiliations:** ^1^Department of Cardiology, Sichuan Mianyang 404 Hospital, Mianyang, China; ^2^School of Nursing, Chengdu Medical College, Chengdu, China; ^3^North Sichuan College of Preschool Teacher Education, Guangyuan, China; ^4^Department of Oncology, Sichuan Mianyang 404 Hospital, Mianyang, China; ^5^School of Basic Medical Sciences, Chengdu Medical College, Chengdu, China

**Keywords:** diabetic peripheral neuropathy, clinical outcomes, musculoskeletal function, exercise, meta-analysis

## Abstract

**Background:**

Diabetes mellitus is a major public health issue, and its complication, diabetic peripheral neuropathy (DPN), can significantly impair foot and ankle joint function, affecting daily activities and quality of life. This systematic review and meta-analysis aimed to evaluate the impact of exercise on musculoskeletal function and clinical outcomes in DPN patients.

**Methods:**

Evaluation of randomized controlled trials (RCTs) of exercise in patients with DPN published from Cochrane Library, PubMed, Embase, Web of Science, Medline, ClinicalKey, CNKI, Wanfang database, VIP Chinese Journal Database, and Chinese Biomedical Literature Database was performed. Revman 5.4 software was used for statistical analysis.

**Results:**

A total of 625 samples were included in 10 studies. It showed that exercise significantly increased ankle dorsiflexion range of motion (SMD = 0.61, 95%CI (0.13, 1.08), *P* < 0.05), ankle flexion range of motion (SMD = 0.59, 95% CI (0.12, 1.06), *P* < 0.05), hallux strength (MD = 1.89, 95% CI (1.00, 2.78), *P* < 0.001), toes strength (MD = 2.51, 95% CI (1.69, 3.33), *P* < 0.001) and lower extremity functional strength (MD = −2.82, 95% CI (−3.88, −1.76), *P* < 0.001), whereas reduced glycosylated hemoglobin (SMD = −1.44, 95% CI (−2.30, −0.57), *P* < 0.01) and body mass index (MD = −0.86, 95% CI (−1.15, −0.57), *P* < 0.001). However, discrepancies were observed between pooled results based on different neuropathy assessment tools.

**Conclusion:**

The available evidence suggests that exercise training is an effective method for improving peripheral neuropathy.

**Systematic review registration:**

https://inplasy.com/, identifier INPLASY202340112.

## 1 Introduction

Diabetes mellitus (DM), as a metabolic chronic disease, has become a growing public health challenge worldwide. According to 2021 data, ~537 million adults aged 20 to 79 years worldwide suffer from diabetes, accounting for 9.3% of the population in this age group, and is expected to increase to 783 million by 2045 (1). Diabetic peripheral neuropathy (DPN) is one of the most common microvascular complications of DM and its prevalence is even higher than that of diabetic retinopathy and diabetic nephropathy (2). Epidemiological studies have shown that about 50% of diabetic patients experience different degrees of DPN during the course of the disease (3), and its prevalence is greatly affected by the study population and diagnostic criteria, ranging from 2.4% to 75.1%, and increases significantly with the age and duration of the disease (4, 5).

The development of DPN is closely associated with long-term hyperglycemia. Persistent hyperglycemia can cause damage such as nerve ischemia and demyelination through various metabolic pathways (such as oxidative stress, polyol pathway, and AGEs accumulation), which can lead to peripheral neurological dysfunction (6). DPN usually has an insidious onset, and the clinical manifestations are mainly symmetrical distal paresthesia, accompanied by decreased muscle strength, hyporeflexia and foot circulation disorders (7). If not intervened in time, it can progress to serious complications such as diabetic foot, which is one of the main causes of foot ulcers and non-traumatic amputations (8). Studies show that DPN increases the risk of foot ulcers by 2–3 times, and its associated complications result in up to $176 billion in direct medical expenses annually, significantly increasing the economic burden on patients and the healthcare system (9, 10). In addition, DPN disrupts neuromuscular coordination as well as proprioceptive function, resulting in dysfunction such as abnormal gait and decreased balance (11, 12). Data have shown that patients with DPN have a significantly higher risk of falls than those without DPN, with a fall incidence of 73%, and are closely associated with fractures, disability, and even death (13, 14).

Given the broad clinical impact of DPN and the heavy health burden it imposes, the development of effective interventions has become a focus of current research attention. However, existing treatment strategies primarily focus on strict glycemic control and relief of neuropathic pain (15), with limited effectiveness in improving functional impairments. With the advancement of the concept of non-pharmacological intervention, exercise training as a safe and feasible treatment has been paid more and more attention. The American Diabetes Association (ADA) (16) also explicitly recommends in its guidelines that diabetic patients engage in various forms of exercise to manage DPN. Studies have shown that exercise can effectively enhance skeletal muscle insulin sensitivity, promote blood glucose regulation (17), and improve muscle strength and proprioceptive function, which can improve the overall functional status of DPN patients to some extent (18, 19). Systematic reviews have now assessed the combined effects of exercise interventions in patients with DPN, but most studies (20–23) have focused on their effects on balance function or gait stability, and integrated evidence for improvement in musculoskeletal function and clinical outcomes remains relatively limited. To address the current evidence gap, this meta-analysis aims to systematically assess the effects of different exercise interventions on musculoskeletal function and clinical outcomes in DPN patients, clarify their potential benefits, and provide evidence-based support for optimizing clinical intervention strategies.

## 2 Methods

This systematic review was conducted in accordance with the Preferred Reporting Items for Systematic Reviews and Meta-Analyses (PRISMA) guidelines (24). The review protocol was registered with the International Platform of Registered Systematic Review and Meta-Analysis Protocols (INPLASY) under the registration number INPLASY202340112.

### 2.1 Search strategy

A comprehensive literature search was conducted in the Cochrane Library, PubMed, Embase, Web of Science, MEDLINE, ClinicalKey, CNKI, Wanfang Data, VIP Database, and the Chinese Biomedical Literature Database, covering publications from database inception to March 2023 in both English and Chinese. Search terms included combinations of subject headings and free-text terms related to the population (“diabetic neuropathies”, “diabetic autonomic neuropathies”, “painful diabetic neuropathies”, “diabetic polyneuropathy”), intervention (“exercise”, “aerobic exercise”, “exercise training”, “physical activity”, “physical exercise”), and study type (“randomized controlled trial”, “RCT”). The detailed search strategy for PubMed is provided in Table 1.

**Table 1:** PubMed searchable.

**Number**	**Search terms**
#1	(“Diabetic Neuropathies” [MeSH Terms]) OR (“Diabetic Neuropath^*^” [Text Word]) OR (“Painful Diabetic Neuropath^*^” [Text Word]) OR (“Diabetic Polyneuropathy” [Text Word]) OR (DPN [Text Word]).
#2	(Exercise [MeSH Terms]) OR (“Exercise Therapy” [Mesh Terms]) OR (“Resistance Training” [Mesh Terms]) OR (“Aerobic Exercise” [Mesh Terms]) OR (“Exercise Training^*^” [Text Word]) OR (“Physical Activit^*^” [Text Word]) OR (“Physical Exercise^*^” [Text Word]).
#3	(“Randomized Controlled Trial” [MeSH Terms]) OR (RCT [Text Word]).
#4	#1 AND #2 AND #3.

### 2.2 Eligibility criteria

The inclusion criteria were as follows: ① study subjects: clearly diagnosed DPN patients, aged ≥18 years. ② interventions: control group received usual healthcare, intervention group received exercise training on the basis of usual healthcare. ③ at least one of the following outcome indicators was included: ankle dorsiflexion range of motion left (ROML), ankle plantar flexion range of motion left (ROML), hallux strength, toes strength, glycated hemoglobin (HbA1c), Michigan diabetic neuropathy score (MDNS), Michigan neuropathy screening instrument (MNSI), five-time sit-to-stand test (FTSST test), body mass index (BMI). ④ randomized controlled trials (RCTs) used.

Exclusion criteria were as follows: ① studies of patients with gestational diabetes. ② literatures that cannot get full text or original data. ③ repeated publications. ④ gray literatures such as conference papers.

### 2.3 Study selection

Two investigators independently screened the literature. Articles with different opinions were arbitrated by a third investigator. Two independent investigators collected and cross-checked the basic information and extracted data. A standardized data extraction form was designed, including author(s)/year, Country, Mean age, Sample size, Duration of diabetes, HbA1c, Exercise program for intervention group, Control group interventions, Follow-up, Outcomes.

### 2.4 Assessment of bias risk

In this study, we used the risk of bias assessment tool recommended by the Cochrane Collaboration Network (25) to assess the risk of bias of the included RCTs. The tool assesses the risk of systematic bias in studies from seven dimensions: randomization method, allocation concealment, blinded implementation, data integrity, selective reporting, and other potential biases. Each dimension was judged as “low risk”, “unclear risk”, or “high risk” based on literature reports. If all dimensions are low risk, the study is rated as low risk overall; if there is at least one high risk dimension, the study is judged as high risk; if there is one or more unclear risks and the remainder are low risk, the study is rated as unclear risk.

### 2.5 Data analysis

In this study, meta-analysis was performed using Review Manager software (RevMan 5.4) as recommended by Cochrane Collaboration. Heterogeneity was assessed by the *I*^2^ statistic, where *I*^2^ values of 25%, 50%, and 75% represented low, moderate, and high heterogeneity (26). If the heterogeneity test results showed *P* ≥ 0.1 and *I*^2^ < 50%, the fixed-effect model was used; if *P* < 0.1 and *I*^2^ ≥ 50%, and the heterogeneity could not be explained by clinical or methodological differences, the random-effects model was used. For the analysis results with high heterogeneity, the possible sources of heterogeneity were further explored through subgroup analysis (with the type of exercise intervention as the grouping variable). When the number of included studies was ≥ 10, Egger's test was used to assess the possibility of publication bias (27). Sensitivity analyses assessed the robustness of the combined results by excluding individual studies one by one. Effect sizes for continuous variables were presented as standardized mean difference (SMD) or weighted mean difference (MD), and 95% confidence intervals (95% CI) were calculated to estimate the precision of the overall effect. The level of heterogeneity was set at α = 0.1, and the remaining tests were set at α = 0.05.

## 3 Results

### 3.1 Literature screening

**Figure 1:**
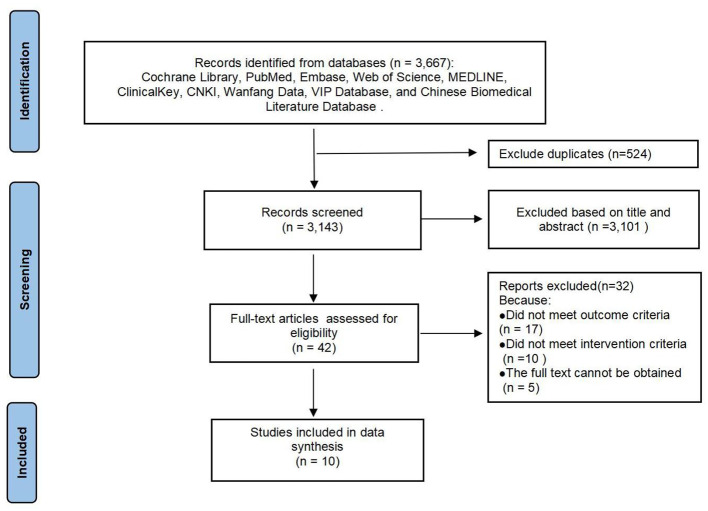
Flowchart of the study selection process.

A total of 3,667 relevant studies were retrieved from the databases. After removing duplicates (*n* = 524) and excluding irrelevant titles and abstracts (*n* = 3,101), 42 studies were selected for full-text review. Among these, 32 studies were excluded due to not meeting the outcome criteria, not meeting the intervention criteria, or the full text being unavailable. Finally, 10 studies were included in the analysis. The study selection process is illustrated in Figure 1.

### 3.2 Characteristics of included studies

The included studies were published between 2013 and 2022, covering six countries. A total of 10 studies were included, comprising seven articles in English and three in Chinese, with a combined sample size of 625 participants (307 in the intervention group and 318 in the control group). The interventions primarily consisted of aerobic and resistance exercises, while other types included tap dance, short-term strength training, and balance training. The frequency of the interventions ranged from two to five times per week, and the duration ranged from 8 to 16 weeks. A brief description of each intervention is provided in Table 2, and detailed information on all included studies is presented in Table 3.

**Table 2:** Short description of interventions included in this review.

**Type of intervention**	**Definition/description**
Foot–ankle therapeutic exercise program (28)	An online foot and ankle exercise program targeting both intrinsic and extrinsic muscles with functional, stretching, and strengthening exercises.
Progressive resistance training (29)	The external resistance is progressively increased over time to promote gradual and sustained improvements in strength.
Supervised resistance training program (30)	All exercise sessions were supervised by an exercise physiologist, with resistance activities adjusted based on real-time blood glucose monitoring.
BioDensity™ Resistance Motion Instrument (Resistance exercise) (31)	Resistance training quantified with the bioDensity™ system.
Medium-intensity elastic resistance exercise (32)	Moderate-intensity elastic resistance training was performed using a yellow resistance band as an exercise aid.
Based on peripheral neuropathy as dominant movement pattern (EPN) (33)	Personalized exercise based on movement impairments caused by nerve damage, such as muscle weakness, sensory loss, and poor balance.
Tapping dance (34)	Striking the floor with tap shoes produces rhythm, strengthens ankle and foot muscles, and stimulates the soles of the feet.
Short-term strength and balance training (19)	An 8-week program of passive joint and muscle strengthening exercises combined with balance training was implemented.
Aerobic exercise (35)	Moderate intensity treadmill exercises.

**Table 3:** Characteristics of the studies included in the meta-analysis.

**Study**	**Country**	**Mean age**		**Sample size**		**duration of diabetes**		**HbA1c (%)**		**Exercise program for intervention group**			**Control group interventions**	**Follow-up (weeks) **	**Outcomes**
		**Intervention group**	**Control group**	**Intervention group**	**Control group**	**Intervention group**	**Control group**	**Intervention group**	**Control group**	**Type of exercise**	**Duration (weeks)**	**Frequency (times/ week)**			
Cruvinel-Júnior et al. (28)	Brazil	56.5 ± 9.9	51.1 ± 10.2	15	15	10.8 ± 7.4	18.8 ± 11.8	—	—	A	12	3	Usual care	6, 12, 24	
Monteiro et al. (36)	Brazil	61.5 ± 11.7	60.1 ± 8.9	15	19	—	—	—	—	A	12	2	Usual care	12, 24	
Khan et al. (29)	Denmark	63 ± 8	63 ± 8	15	15	10 ± 8	10 ± 8	7.40 ± 1.20	7.40 ± 1.20	B1	12	2 or 3	Usual care	12	
Gholami et al. (30)	Ireland	63 ± 3	64 ± 3	15	14	—	—	9.09 ± 1.82	9.97 ± 1.82	B2	12	3	Usual care	12	
Luo et al. (33)	China	56.25 ± 2.89	55.53 ± 3.80	40	40	11.16 ± 2.26	11.33 ± 2.85	8.37 ± 0.67	8.63 ± 1.16	C	12	—	Educational workshops	12	
Zhao et al. (34)	China	63.4 ± 3.55	66.6 ± 5.81	20	20	7.81 ± 4.86	7.92 ± 3.16	6.31 ± 1.21	6.86 ± 0.616	D	16	3	Usual care	16	
Yang et al. (31)	China	59.28 ± 10.18	59.52 ± 8.92	50	50	—	—	7.13 ± 1.04	6.62 ± 1.21	B3	24	4	Usual care	12, 24	
Hu and Zhang (32)	China	75.2 ± 0.5	75.8 ± 0.6	45	45	—	—	8.36 ± 0.25	8.35 ± 0.21	B4	12	3	Usual care	12	
Venkataraman et al. (19)	Singapore	62	62	67	67	15.3 ± 10.7	15.3 ± 10.7	8.5	8.5	E	8	3	Foot care education	8, 24	
Dixit et al. (35)	India	54.40 ± 1.24	59.45 ± 1.16	29	37	5.46 ± 0.16	6.84 ± 0.14	—	—	F	8	5 or 6	Usual care	8	

Note: “—”, not reported; A, Foot–ankle therapeutic exercise program; B1, Progressive resistance training; B2, Supervised resistance training program; B3, BioDensity ™ Resistance Motion Instrument (Resistance exercise); B4, Medium-intensity elastic resistance exercise; C, Based on peripheral neuropathy as dominant movement pattern (EPN); D, Tapping dance; E, Short-term strength and balance training; F, Aerobic exercise; , Ankle dorsiflexion range of motion left; , Ankle plantar flexion range of motion left; , Hallux strength; , Toes strength; , glycosylated hemoglobin (HbA1c); , Michigan Diabetic Neuropathy Score (MDNS); , Michigan Neuropathy Screening Instrument (MNSI); , five-time sit-to-stand test (FTSST); , body mass index (BMI).

### 3.3 Quality of the evidence

**Figure 2:**
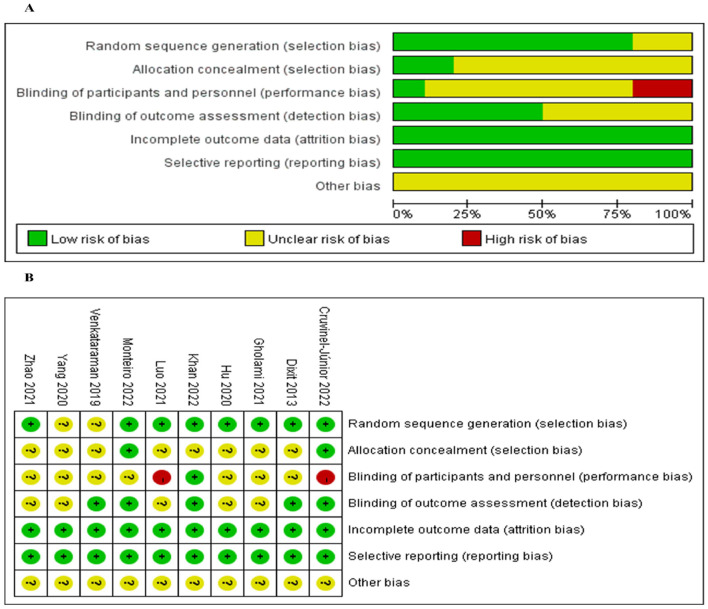
**(A)** Bias risk assessment of the included studies. **(B)** Summary of bias risks of the included studies.

Of the 10 included studies, eight studies reported using random number table or lottery to generate random sequences, and the remaining two studies only mentioned randomization and did not describe specific random implementation methods. Two studies were stored in opaque, sequentially numbered, sealed envelopes using an allocation order. Five studies mentioned blinding of outcome assessors, two mentioned that study reports could not blind study subjects and interventions, and the remaining studies did not mention blinding of subjects, researchers, or outcome assessors. All studies described losses to follow-up and withdrawals in detail and reported pre-specified outcome measures. None of the investigators mentioned other risk of bias situations, such as conflicts of interest. The Cochrane risk of bias summary and graph are presented in Figures 2A, B, respectively.

### 3.4 Heterogeneity analysis of HbA1c

**Figure 3:**
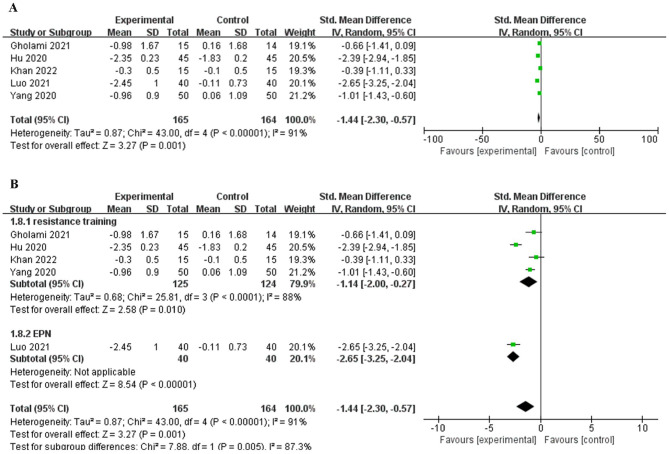
Forest plot and subgroup analysis of HbA1c. **(A)** Overall analysis. **(B)** Subgroup analysis.

A total of five studies (29–33) included HbA1c as the outcome measure. The heterogeneity test showed that there was great heterogeneity among the studies *(P* < 0.001, *I*^2^ = 91%). The random-effects model was used to combine the effect size. The results (Figure 3A) showed that the intervention group was superior to the control group in reducing HbA1c [SMD = −1.44, 95% CI (−2.30, −0.57), *P* < 0.01]. Sensitivity analysis of included studies revealed little change, suggesting good stability of the results. However, the final results remained statistically significant (*P* < 0.01), and the intervention group was superior to the control group in reducing HbA1c.

In the subgroup analysis (Figure 3B), the heterogeneity of resistance exercise was statistically significant among four studies (*P* < 0.001, *I*^2^ = 88%), and significant difference detected between the resistance exercise and control groups [SMD = −1.14, 95% CI (−2.00, −0.27), *P* < 0.05]. One study reported Based on peripheral neuropathy as dominant movement pattern (EPN), the heterogeneity was not applicable. Overall, there was a difference between the subgroups (*P* < 0.01, *I*^2^ = 87.3%), indicating that the type of exercise was one of the sources of heterogeneity.

### 3.5 Heterogeneity analysis of BMI and ankle ROML

**Figure 4:**
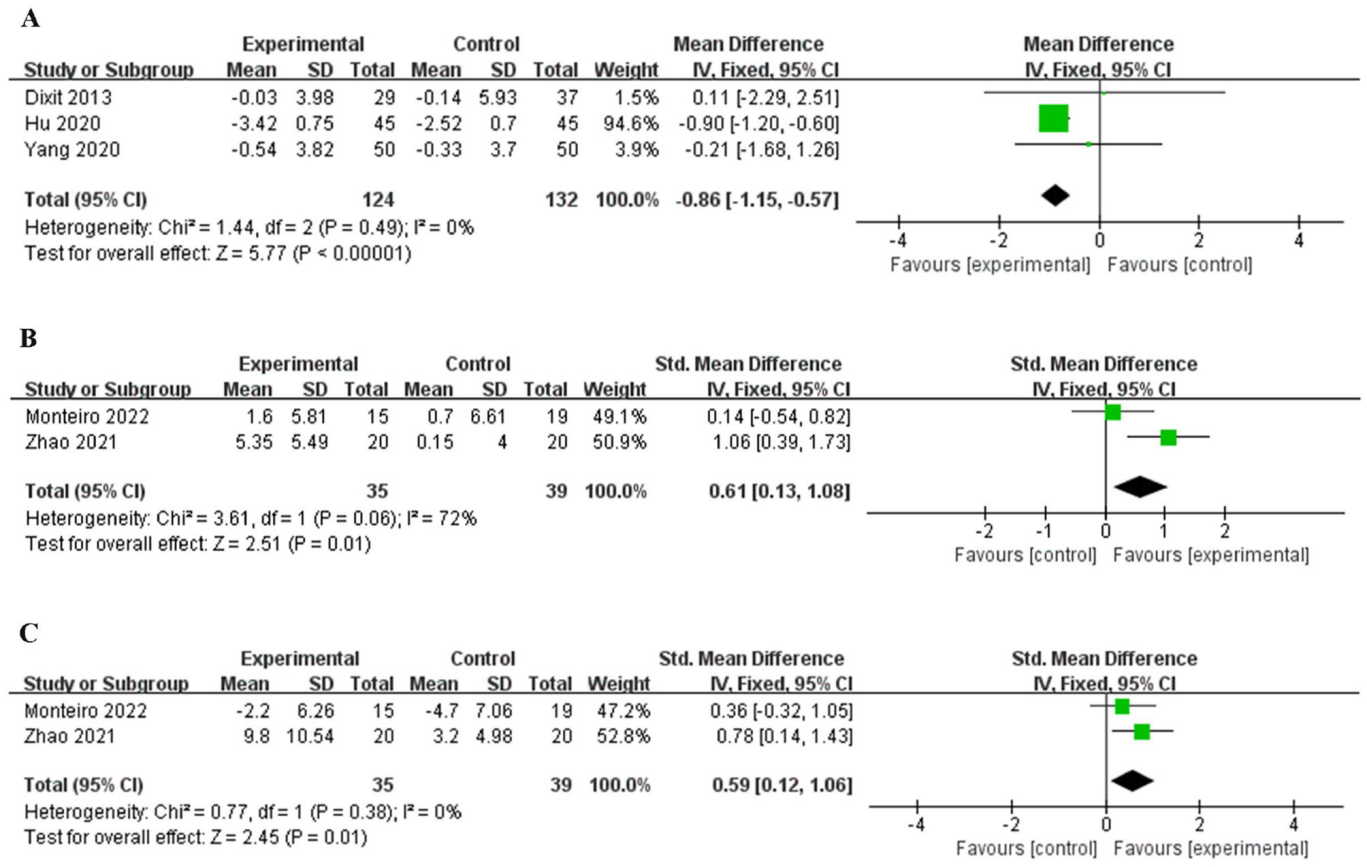
Forest plot of BMI and ankle ROML. BMI **(A)**, ankle dorsiflexion ROML **(B)**, ankle plantar flexion ROML **(C)**.

BMI was included as an outcome measure in three studies (31, 32, 35), and there was no statistically significant difference in heterogeneity (*P* = 0.49, *I*^2^ = 0%, Figure 4A). The fixed effects model combined effect size showed that the intervention group was superior to the control group in reducing BMI [MD = −0.86, 95% CI (−1.15, −0.57), *P* < 0.001].

Two studies (34, 36) included ankle dorsiflexion ROML as the outcome measure. The heterogeneity test showed that there was great heterogeneity among the studies (*P* = 0.06, *I*^2^ = 72%). The results (Figure 4B) showed that the intervention group was superior to the control group in increasing ankle flexion range of motion [SMD = 0.61, 95% CI (0.13, 1.08), *P* < 0.05].

Two studies (34, 36) included a ankle plantar flexion ROML, but there was no statistically significant difference in heterogeneity (*P* = 0.38, *I*^2^ = 0%, Figure 4C). The random-effects model combined effect size showed that the intervention group was superior to the control group in increasing ankle extension range of motion [SMD = 0.59, 95% CI (0.12, 1.06), *P* < 0.05].

### 3.6 Heterogeneity analysis of hallux and toes strength and FTSST test

**Figure 5:**
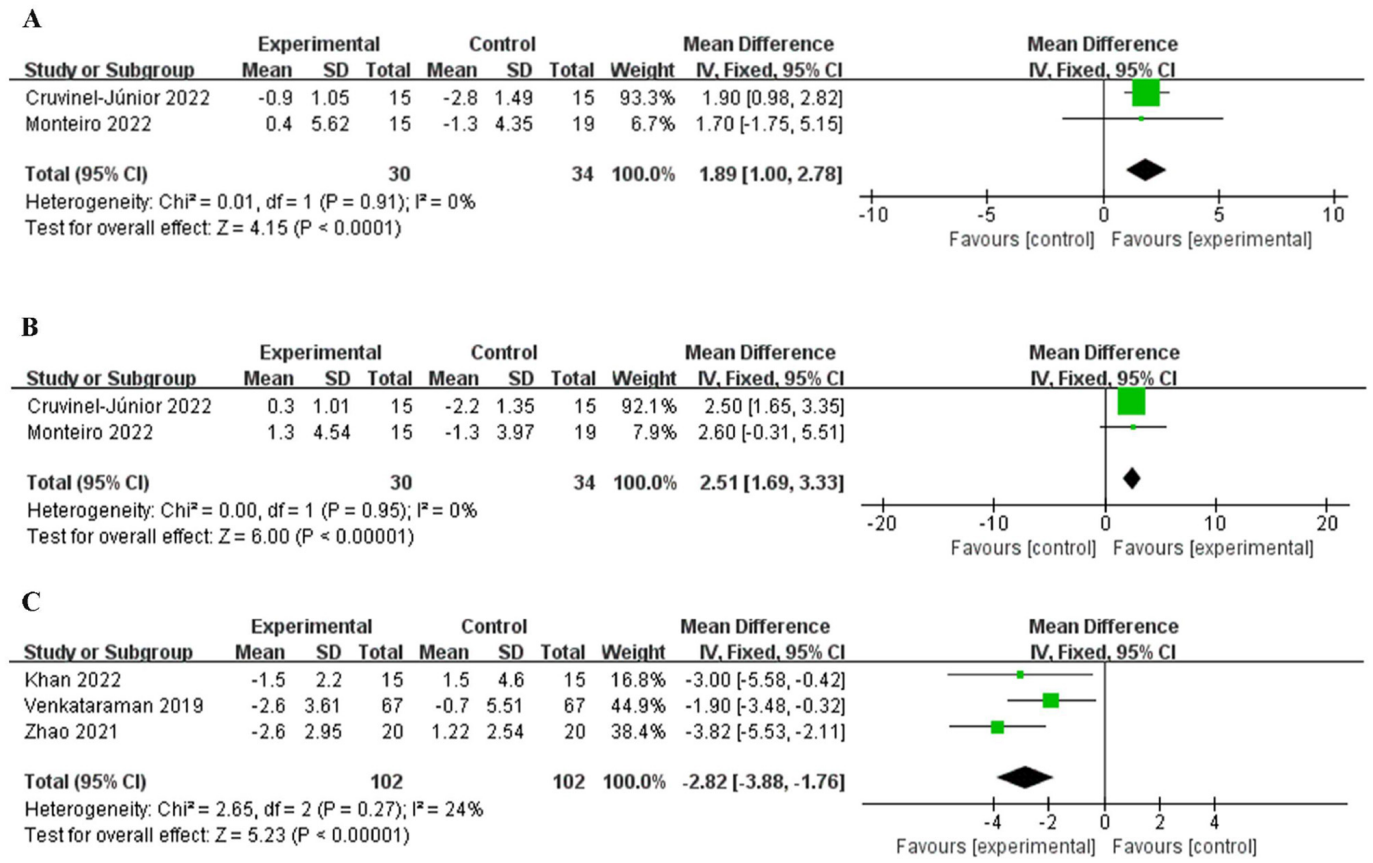
Forest plot of hallux and toes strength and FTSST test. Hallux strength **(A)**, Toes strength **(B)**, FTSST test **(C)**.

Two studies (28, 36) included hallux strength and toes strength, and there was no statistically significant difference in heterogeneity between studies (Figures 5A, B). The fixed effects model combined the effect size showed that the intervention group was superior to the control group in increasing hallux strength [MD = 1.89, 95% CI (1.00, 2.78), *P* < 0.001] and toes strength [MD = 2.51, 95% CI (1.69, 3.33), *P* < 0.001].

Three studies (19, 29, 34) included FTSST test, and there was no statistically significant difference in heterogeneity between studies (*P* = 0.27, *I*^2^ = 24%, Figure 5C). The fixed effects model combined the effect size showed that the intervention group was superior to the control group in enhancing lower extremity functional strength [MD = −2.82, 95% CI (−3.88, −1.76), *P* < 0.001].

### 3.7 Heterogeneity analysis of MNSI and MDNS

**Figure 6:**
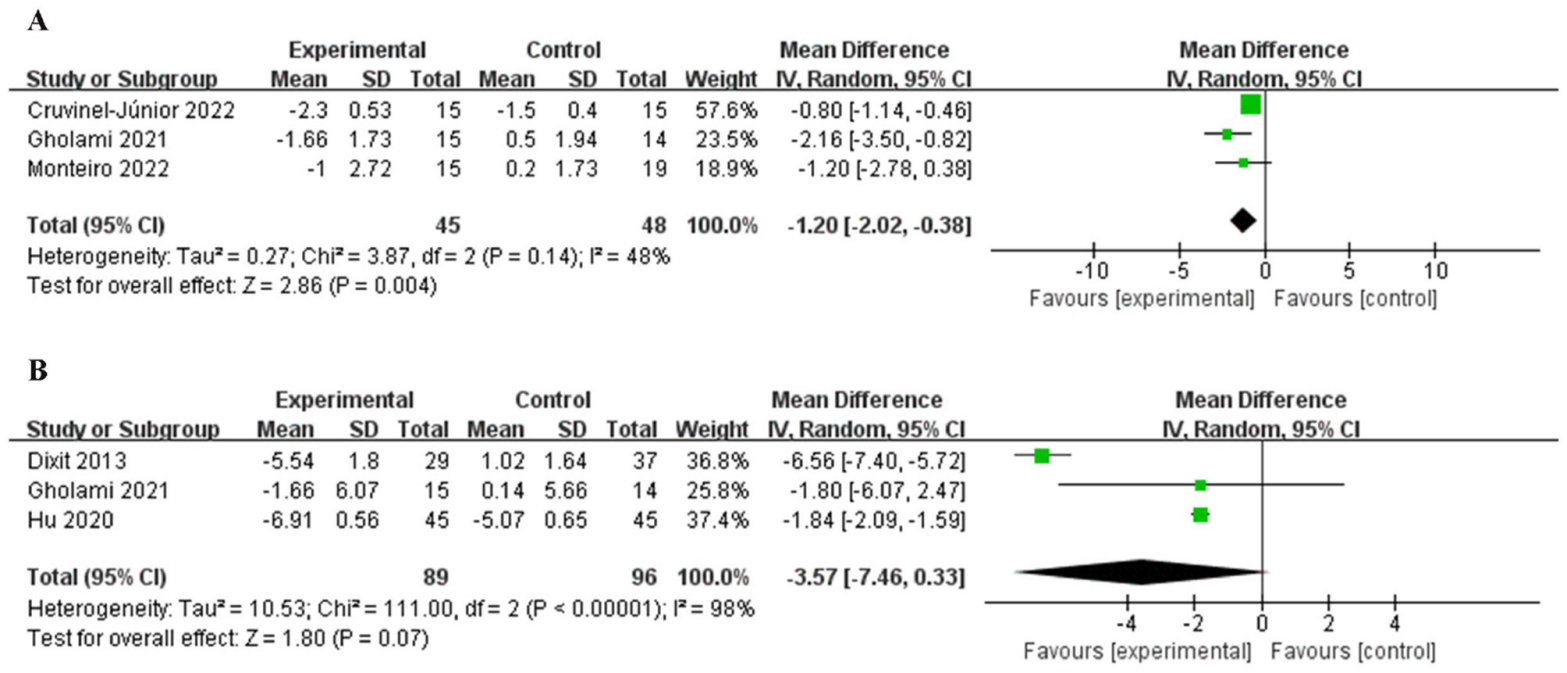
Forest plot of MNSI and MDNS. MNSI **(A)**, MDNS **(B)**.

Three studies (28, 30, 36) used MNSI as an outcome measure, and there was no statistically significant difference in heterogeneity (*P* = 0.14, *I*^2^ = 48%, Figure 6A). The fixed effects model combined the effect size showed that the intervention group was superior to the control group in improving peripheral neuropathy [MD = −1.20, 95% CI (−2.02, −0.38), *P* < 0.01].

Other three studies (30, 32, 35) used MDNS as an outcome measure, and there was a statistically significant difference in heterogeneity (*P* < 0.001, *I*^2^ = 98%, Figure 6B). However, there was no statistically significant difference between groups in improving peripheral neuropathy [MD = −3.57, 95% CI (– 7.46, 0.33), *P* = 0.07].

## 4 Discussion

This meta-analysis represents a comprehensive synthesis of data currently available for DPN intervention studies. We evaluated 10 randomized controlled trials involving nine different exercise interventions. This systematic review and meta-analysis quantitatively assessed whether exercise training can improve musculoskeletal function and clinical outcomes in DPN patients. The findings indicate that exercise training combined with standard care has a positive effect on improving ankle joint range of motion, hallux and toe strength, and lower limb functional strength, while also reducing HbA1c and BMI levels. Exercise training may have a positive effect on reducing MNSI scores in improving neurological symptom scores, but the existing evidence cannot yet support that exercise training has a clear advantage on MDNS scores.

### 4.1 Musculoskeletal functional (Ankle ROM, hallux and toes strength, FTSST)

This study showed that exercise intervention, especially ankle joint and distal lower limb muscle training, significantly improved ankle range of motion, toe and lower limb muscle strength in patients with DPN. Meta-analysis of two studies showed that both foot and ankle training and tap dance interventions significantly increased ankle dorsiflexion and plantar flexion range of motion, consistent with previous systematic reviews supporting the effectiveness of physical therapy targeting the foot and ankle in improving joint flexibility (37). The possible mechanism is that this type of training extends joint range of motion by applying mechanical stimulation to the soft tissues around the ankle joint and enhancing its flexibility and neuromuscular control (38). Meta-analysis of two other studies showed that foot and ankle training significantly enhanced hallux and toe muscle strength in patients with DPN, with low heterogeneity among studies, suggesting that it has a consistent effect in enhancing distal muscle strength. This is consistent with the findings of Prókai et al. (39). The latter points out that DPN patients are often accompanied by decreased isokinetic torque of the ankle joint and foot muscle atrophy, especially significantly weakened flexor hallux and minor muscle groups (40). Muscle strength of the hallux and phalanges is key to maintaining gait stability and preventing falls, and its enhancement helps to improve weight-bearing control and dynamic balance, thereby improving overall functional mobility (41).

In addition, a meta-analysis of three studies showed that different types of exercise interventions significantly improve functional strength level of the lower limbs in DPN patients. Among them, progressive resistance training promotes muscle protein synthesis and reverses muscle fiber atrophy by activating the mTOR-p70S6K pathway, delaying muscle loss and enhancing muscle strength more effectively than low-intensity training (42, 43); strength and balance training helps to improve knee extensor strength, motor ability and walking performance (44, 45); while tap dance training improves coordination and balance control ability in addition to enhancing lower limb muscle strength (46). Cruvinel-Júnior et al. (47) further pointed out that foot and ankle exercise training can not only directly improve foot function, but also promote functional recovery by indirect mechanisms such as relieving pain and increasing joint mobility. In summary, exercise intervention, especially foot and ankle training, is effective in improving lower limb function in patients with DPN and can be used as a clinically important rehabilitation strategy.

### 4.2 Clinical outcomes (BMI, HbA1c)

The clinical manifestations of DPN can be reflected by BMI and HbA1c. This study shows that exercise intervention significantly improved BMI as well as HbA1c in DPN patients. Numerous studies have demonstrated that exercise interventions effectively promote body fat reduction with weight management in different populations (48, 49). This meta-analysis further complements the evidence-based support of exercise for BMI improvement in DPN patients. Exercise lowers BMI by increasing energy expenditure and optimizing metabolic function, particularly by enhancing skeletal muscle insulin sensitivity, promoting lipid metabolism, and reducing fat accumulation (50). The incidence and severity of diabetic neuropathy are positively correlated with the duration of hyperglycemia and blood glucose levels (51), and persistent or fluctuating hyperglycemia can induce irreversible peripheral nerve damage (52). Therefore, active glycemic control is essential for diabetes and its complications, particularly DPN. In this paper, the heterogeneity of different studies of HbA1c is large, but there are statistical differences between the intervention group and the control group, and the intervention group is superior to the control group, which is consistent with the results of previous studies (53, 54). Both EPN and resistance exercise had good effects on improving HbA1c by DPN. Structured resistance exercises >150 min per week in patients with T2DM reduced HbA1c by 0.89% (55). An animal study (56) showed that exercise regulates the metabolic system of the body, and lowers blood glucose levels, thereby reducing the degree of microvascular injury, strengthening nerve conduction function, and ultimately improving peripheral neuropathy symptoms. Therefore, the effect of intensive glycemic control on improving DPN is positive, and the more stringent the glycemic control, the more patients benefit.

### 4.3 Neuropathy symptom scores (MNSI, MDNS)

MNSI and MDNS are currently among the most commonly used screening tools for assessing DPN. Although studies have shown a significant reduction in the development of abnormal neurological examinations in patients undergoing exercise intervention (57), and improvements in neurological function may be attributed to the strengthening of existing sensory-motor pathways (58), the combined results of this meta-analysis are not fully consistent with previous studies. In the three studies using the MNSI score, the combined results showed a significant advantage of exercise training over usual care in improving neuropathy symptoms. However, in the three studies using the MDNS score, the results did not reach statistical significance and did not clearly indicate a significant effect of exercise training on this scale. The inconsistency in the above results may reflect multiple differences in study populations, intervention protocols, outcome setting, and bias control. Among them, MNSI mainly assesses subjective symptoms and some physical parameters, which are suitable for early screening and are greatly subjectively affected; MDNS focuses on objective neurological function such as muscle strength, reflexes and pain sensation, which is suitable for moderate to severe DPN with more stable results, but requires high study design (59). Although the results of this study suggest that exercise training may help improve neurological function in some indicators, the relevant evidence is still insufficient. In the future, more rigorous, high-quality randomized controlled trials should be conducted in combination with DPN grading and objective indicators (e.g., nerve conduction velocity) to determine the optimal exercise intervention program.

### 4.4 Strengths and limitations

In this study, a detailed search strategy was employed by searching different types of databases and trial registries. In addition, heterogeneity among studies of most outcome measures was low in comparison to different outcome measures, which indicated that the reliability of this meta-analysis was high. We try to explain the source of heterogeneity through sensitive analysis and subgroup analysis, so that the study results are more persuasive. In addition, we evaluated the effect of exercise training on improving DPN through a meta-analysis with a higher evidence-based level, suggesting that it can improve musculoskeletal function and clinical outcome measures in patients. It can provide clinicians with evidence to help them make better clinical decisions.

However, this study still has several limitations, and caution should be exercised when interpreting the results. First, all included studies lacked long-term follow-up data, and the sustained efficacy of exercise training for DPN remains unclear. Additionally, some studies failed to complete routine follow-up due to the impact of the COVID-19 epidemic, limiting a comprehensive assessment of long-term effects. Second, this study covers a variety of exercise intervention modalities, but most of them are single-center studies with small sample sizes, and there are some limitations in the representativeness and external generalizability of the results. Third, the included studies differed in terms of type of exercise, duration and frequency of intervention, which may lead to some degree of clinical heterogeneity. Finally, some studies lacked sufficient reporting on methodological details such as concealment of random assignment, blinded implementation, and outcome measurement, which may have biased the meta-analysis results. In the future, it is urgent to design standardized large-sample, multicenter randomized controlled trials to verify and expand the findings of this study.

## 5 Conclusion

Our meta-analysis suggests that exercise interventions, particularly foot and ankle training, contribute to improving DPN related musculoskeletal dysfunction and clinical outcomes compared to usual care measures. These results will provide a basis for adjuvant treatment strategies and clinical decision-making in DPN.

## 6 Relevance for clinical practice

The American College of Sports Medicine (17) recommends that patients with diabetes, particularly those with DPN, engage in muscle-strengthening exercises 2–4 times per week. However, nurses may encounter challenges in selecting appropriate exercise interventions for patients with DPN, which may affect patients' achievement of optimal treatment outcomes. A better understanding of the latest evidence-based information and potential risks and benefits of exercise interventions for DPN patients is essential for caregivers to optimize clinical decision-making and improve individualized services. In this study, we summarized the effects of structured exercise intervention on musculoskeletal function and clinical outcomes in patients with DPN in recent years through systematic review and meta-analysis, and the results showed that this intervention had a significant effect in improving ankle range of motion, hallux and toe strength, and lower limb muscle strength, and could effectively reduce HbA1c and BMI levels. These findings emphasize the necessity of integrating evidence-based exercise prescriptions into routine care, providing practical intervention directions for nursing practice. Due to heterogeneity in intervention effects across different studies, it is particularly important to develop individualized exercise programs and implement them through a multidisciplinary team comprising rehabilitation therapists, diabetes management specialists, and dietitians. In clinical practice, the effectiveness of exercise interventions should be continuously assessed and dynamically adjusted to ensure long-term patient adherence and achieve optimal treatment outcomes. In addition, future studies should further evaluate the cost-effectiveness and accessibility of exercise interventions and consider the impact of cultural, economic, and psychological factors on patient acceptance. Incorporating patient preferences into intervention design can enhance adherence and promote feasibility.

## Data Availability

The original contributions presented in the study are included in the article/supplementary material, further inquiries can be directed to the corresponding authors.
